# Differential Expression of MSTN Isoforms in Muscle between Broiler and Layer Chickens

**DOI:** 10.3390/ani12050539

**Published:** 2022-02-22

**Authors:** Dong-Hwan Kim, Young Min Choi, Joonbum Lee, Sangsu Shin, Sanggu Kim, Yeunsu Suh, Kichoon Lee

**Affiliations:** 1 Department of Animal Sciences, The Ohio State University, Columbus, OH 43210, USA; kim.4094@osu.edu (D.-H.K.); ymchoi1@knu.ac.kr (Y.M.C.); lee.3920@osu.edu (J.L.); sss@knu.ac.kr (S.S.); suh.83@osu.edu (Y.S.); 2 Department of Animal Sciences, Kyungpook National University, Sangju 37224, Korea; 3 Interdisciplinary Ph.D. Program in Nutrition, The Ohio State University, Columbus, OH 43210, USA; 4 Department of Animal Biotechnology, Kyungpook National University, Sangju 37224, Korea; 5 Department of Veterinary Biosciences, College of Veterinary Medicine, The Ohio State University, Columbus, OH 43210, USA; kim.6477@osu.edu

**Keywords:** myostatin isoforms, chicken, broiler, layer, muscle hypertrophy

## Abstract

**Simple Summary:**

Increasing the growth rate of food animals with a concomitant increase in muscle yield is a primary goal of animal producers. The identification of factors and a better understanding of the underlying mechanisms of these factors for muscle growth are important if we are to improve meat production in domestic animals. In avian species, MSTN-A has anti-myogenic activities and MSTN-B functions as a pro-myogenic factor. In this study, the expression of the *Mstn* isoforms and the sizes of myofibers and muscle bundles during development were analyzed in broiler and layer chicken breeds. Although the expression levels of total *Mstn* were not different between the two breeds, the ratios of *Mstn-B* to *-A* were significantly higher in the broilers with muscle hypertrophy and hyperplasia compared to the layers. Further characterizations of the muscle in broilers revealed a greater bundle area but similar fiber number per bundle compared to the layers. Our study demonstrated higher ratios of *Mstn-B*/*-A* expression in the muscles of broiler chickens, suggesting that the development of strategies to enhance the expression of Mstn-B will lead to increased muscle growth and poultry production.

**Abstract:**

Myostatin (Mstn)-A, the main isoform among Mstn splicing variants, functions as a negative regulator, whereas Mstn-B functions as a positive regulator in muscle development. Because broiler chickens are a fast-growing breed raised for meat production and layer chickens are a slow-growing breed raised for egg production, differences in the expression of *Mstn* isoforms between the two distinct breeds were analyzed in this study. There was no difference in the expression levels of total *Mstn* (*Mstn-A* and *-B* forms) during embryonic development and at D33 between the two breeds. Interestingly, the ratios of *Mstn-B* to *-A* were significantly higher in the broiler compared to the layer at most ages. In pectoralis major muscle (PM) tissue, the cross-sectional area (CSA) of muscle fiber was significantly greater in the broiler. The broiler also showed greater bundle CSA and a similar fiber number per bundle compared to the layer at D5 and D33. These data suggest that the greater bundle CSA with myofiber hypertrophy in the broilers is associated with greater muscle growth. The relationship between the expression of *Mstn* isoforms and growth rate can be used as a potential genetic marker for the selection of higher muscle growth in chickens.

## 1. Introduction

The chicken market has been steadily increasing during recent decades in the United States. To meet the demands of consumers, geneticists have selected for chickens that are fast growing with a high body weight and meat yield. Although for decades, genetic selection has been successful in establishing broiler and layer chickens to produce meat and eggs, respectively, the genetic factors contributing to these distinctive characteristics between broilers and layers have not been fully studied.

Myostatin (MSTN), also known as growth/differentiation factor-8 (GDF-8), is mainly expressed in skeletal muscle. Its negative regulatory effects on muscle growth were demonstrated previously, where inactivation of MSTN resulted in increased muscle mass in animals and humans [1,2,3]. In addition, targeted genome edition in the MSTN gene resulted in a 30% increase in the muscle weight in quail [4] and significantly increased the growth rate in chickens [5].

In avian species, alternative splicing of Mstn resulted in the production of five isoforms (Mstn-A to -E forms) in various tissues, and Mstn-A and -B are the dominantly expressed forms in muscle tissues [6]. Our previous study revealed that the MSTN-A form suppressed myogenic differentiation but the MSTN-B form promoted myogenic differentiation of the quail myoblast cell line (QM7) [6]. In addition, overexpression of MSTN-B in quail resulted in increased muscle weight with muscle hyperplasia [7]. These in vitro and in vivo studies support a pro-myogenic function of MSTN-B in avian species, the opposite of MSTN-A, which has a conventional anti-myogenic function. Therefore, the objective of this study was to determine to what extent broilers and layers show temporal changes in the total amounts of MSTN and/or ratios of MSTN-B/-A in the muscle.

## 2. Materials and Methods

### 2.1. IACUC Approval and Animal Source

All the animal care and experiments were approved by The Ohio State University Institutional Animal Care and Use Committee (protocol no. 2020A00000094). Fertile eggs of broilers (a cross of Hubbard M99 (male) and Cobb 500FF (female)) and layers (Hy-Line white leghorn) were kindly donated by the Cooper Hatchery, Oakwood, OH, USA, and the Hy-Line North America, LLC, Warren, IN, USA, respectively. The eggs were incubated with turning through a 90-arc rotation every 2 h and all animals had free access to feed and water after hatching. Pectoralis major muscle (PM) tissues were sampled at different ages: embryonic day (E) 15 and 17, and post-hatch day (D) 5 and 33. Collected samples were immediately frozen in liquid nitrogen. They were placed in a −80 °C freezer until use for RNA extraction or fixed in 10% neutral buffered formalin for 48 h for histological analysis. Chickens were sacrificed by CO_2_ inhalation followed by cervical dislocation as guided by the IACUC protocol.

### 2.2. Quantitative Real-Time PCR (qPCR) and Semi-Quantitative PCR (sqPCR)

**Figure 1:**
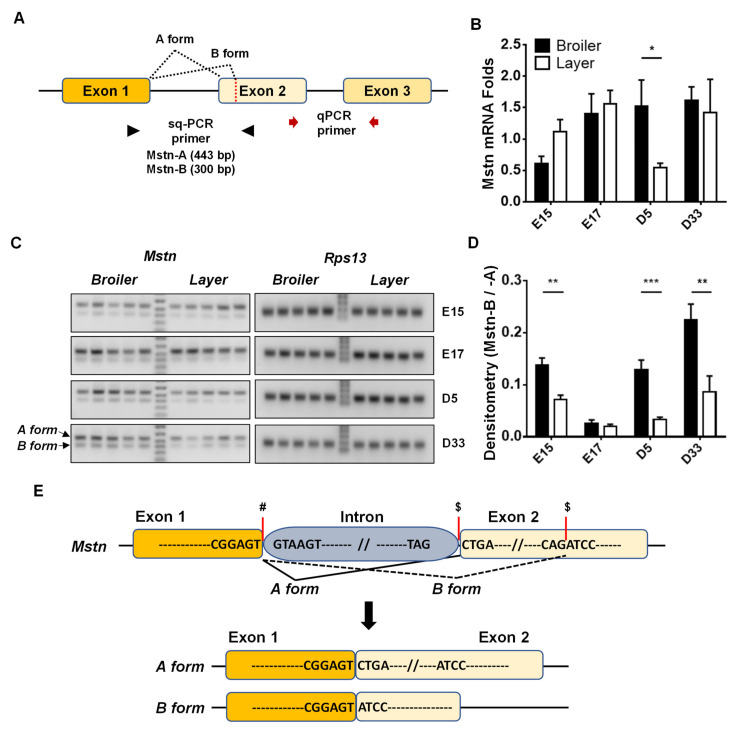
Comparisons of the expression levels of *myostatin* (*Mstn*) isoforms in broiler and layer chickens. (**A**). The schematic diagram shows the location of specific primer sets for qPCR and sqPCR of the chicken *Mstn* gene. (**B**). Quantitative analysis of the *Mstn* expression levels during development by qPCR (n = 5). (**C**,**D**). Gel electrophoresis of sqPCR products of *Mstn* and densitometry analyses. sqPCR products were separated by gel electrophoresis and the densitometry analyses (ratios of *Mstn-B* form to *Mstn-A* form) were performed using NIH ImageJ software. *Rps13* was used as an internal control for qPCR and sqPCR. The multiple *t*-test was used for statistical analysis of the broiler and layer chickens by the Graphpad PRISM 6.02 program. The values presented are means ± SEM (n = 5). *: *p* < 0.05, **: *p* < 0.01 and ***: *p* < 0.001. Abbreviations: E, embryonic day; D, postnatal day. (**E**). Alternative splicing (AS) events of *Mstn* and sequences of splicing junctions. #: alternative 5′ splice site, $: alternative 3′ splice site.

Total RNA isolation and cDNA synthesis were followed in our previous study [8], and then cDNA was used for both qPCR and sqPCR. To quantify the expression levels of the combined *Mstn-A* and *-B* form, specific primer sets were designated to be located in common sharing exons, exon 2 and exon 3 of the chicken Mstn genome, as shown in Figure 1A. qPCR was performed in a thermo cycler, the ABI 7500 (Applied BioSystems, Foster City, CA, USA) using AmpliTaq Gold polymerase (#N8080241, Applied BioSystems) with the following conditions: 95 °C for 10 min followed by 40 cycles of 94 °C for 15 s, 55 °C for 45 s, 72 °C for 40 s, and 82 °C for 32 s with the following primer sets: *Mstn*, F: 5’-GGTATCTGGCAGAGTATTGATGTGAA and R: 5’-ACGGTCCCGCAGAGATTTTG, and *Ribosomal Protein S13* (*Rps13*), F: 5′-AAGAAGGCTGTTGCTGTTCG and R: 5′-GGCAGAAGCTGTCGATGATT. SYBR green I was used as the detection dye for qPCR. *Rps13* was used as the internal control to normalize and analyze the data using the ddCt method [9].

As a traditional and convenient method to determine the relative incidence of two splice variants [10], sqPCR was performed to measure changes in the *Mstn* variants (*Mstn-A* and *-B* form) during development using DNA Taq-polymerase (#M0320, New England BioLabs, Ipswich, MA, USA). The sqPCR conditions were 30 cycles for denaturing at 95 °C for 30 s, annealing at 52 °C for 30 s, and extension at 72 °C for 30 s with specific primer sets, which can amplify both *Mstn-A* and *-B* forms: F: 5′-A GAAAAAGACGGACTGTGCAATG and R: 5′- GCCAATTTTGCAGCACTGTC. To visualize and directly compare the splice isoforms between broiler and layer chickens on each developmental day, the sqPCR products were separated in 1 1.5% agarose gel by electrophoresis and imaged by a gel imaging system (Azure C600, Azure Biosystems, CA, USA). Then, the densities of 2 different sizes of the PCR products (443 bp for *Mstn-A* form or 300 bp for *Mstn-B* form) were analyzed to calculate the relative expression levels of the splice isoforms (*Mstn-B* / *Mstn-A*) in PM tissues by densitometry analysis using NIH ImageJ software. *Rps13* was used as an internal control for sqPCR. The loci for the primers of *Mstn* in the chicken genome are shown in Figure 1A.

### 2.3. Analysis of MSTN Alternative Splicing in Chicken

To analyze the exonic or adjacent intronic sequences of the *Mstn-A* and *-B* form in the broiler and layer chicken breeds, genomic DNA (gDNA) was extracted from 6 broiler and layer chickens. PCR was performed to amplify exon 1 to exon 2 of the chicken Mstn genome using LA Taq DNA polymerase (#RR042, Takara, San Jose, CA, USA). PCR products were amplified for 35 cycles for denaturing at 93 °C for 30 s, annealing at 55 °C for 30 s, and extension at 68 °C for 4 min with specific primer sets: F: 5′-A GAAAAAGACGGACTGTGCAATG and R: 5′-GCCAATTTTGCAGCACTGTC. The PCR products were sequenced at The Ohio State University Core Facility and the consensus sequences of the alternative splicing (AS) junction were identified by analyzing the sequencing data.

### 2.4. Histological Processing and Measurement of the Muscle Cell Size

Fixed PM tissues at D5 and D33 were processed for paraffin embedding and then cut into 5 μm sections. To measure the muscle cell size, the sections were stained with hematoxylin and eosin (HE) after de-paraffinization in xylene and re-hydrated in serially diluted ethanol. Stained slides were imaged under a microscope (EVOS cell imaging system, ThermoFisher Scientific, Waltham, MA, USA). ImageJ software (NIH ImageJ 1.52 s, http://imagej.nih.gov/ij (accessed on 21 January 2021)) was used to determine the muscle cell size and numbers. The average of the cross-sectional area (CSA) of PM tissues was calculated by dividing randomly selected areas by the total number of cells within the area.

### 2.5. Statistical Analysis

All data were expressed as means ± SEM (n ≥ 5). The data was analyzed using Graphpad Prism software, version 6.02. For all comparisons in this study, multiple *t*-tests were conducted. *p*-value of *p* < 0.05 was considered a statistically significant difference.

## 3. Results

### 3.1. Expression Levels of Mstn Isoforms in Broiler and Layer Chickens

The mRNA expression levels of *Mstn* showed no significant difference between broiler and layer chicken breeds at E15, E17, and D33, except at D5 (broiler vs. layer, 1.53 ± 0.41 vs. 0.55 ± 0.07, *p* < 0.05 (Figure 1B)). However, our previous study proved that avian species have two major isoforms of *Mstn* (*Mstn-A* and *-B*) in muscle, unlike mammals, which only one form (MSTN-A) [6]. Given the anti-myogenic and pro-myogenic effects of the Mstn-A and -B forms, respectively, in avian species [6,7], the ratios of *Mstn-A* and *-B* can provide more informative data that might help in relating the different growth characteristics of muscle in the two chicken breeds. To do this, sqPCR was performed to amplify both isoforms, which were then separated into 2 different sizes (547 bp for the *A* form and 404 bp for the *B* form) in agarose gel (1.2%) electrophoresis to present the expression of the *Mstn-A* and *-B* variants at different ages. The ratios of *Mstn-A* and *MSTN-B* were assessed based on densitometry analysis of the specific bands presented in the gel pictures (Figure 1C,D). This study clearly showed higher ratios of the *B/A* form expression in broilers than in layers at most time points (broiler vs. layer, 13.75 ± 1.42 vs. 7.21 ± 0.78, *p* < 0.01 at E15, 12.90 ± 1.88 vs. 3.32 ± 0.46, *p* < 0.001 at D5, and 22.46 ± 3.07 vs. 8.68 ± 3.00, *p* < 0.01 at D33 (Figure 1C,D)). 

Our analyses of the sequencing data for chicken *Mstn* cDNA suggest that the generation of *Mstn* AS isoforms originated from the alternative 3′ splice sites rather than the alternative 5′ splice site [6]. To investigate whether sequence variations in the 3′ splice sites for the *Mstn-A* and *-B* forms can explain the alternative splicing of *Mstn* and different ratios of *Mstn-B/-A* expression between broiler and layer chickens, sequencing analysis was performed on the chicken *Mstn* genome. Comparisons of the AS isoforms of the *Mstn* gene in broiler and layer chicken breeds revealed that the 3′ acceptor sites for both the *Mstn-A* and *-B* forms are TAG/CTGA and CAG/ATCC, respectively (Figure 1E). These sequences were identical between the two chicken breeds and matched with the consensus sequences (NAG/NN) of 3′ acceptor sites, which may allow both isoforms to be generated by AS mechanisms. 

### 3.2. Histological Comparisons of Pectoralis Major Muscle at D5 and D33 in Broiler and Layer Chickens

**Figure 2:**
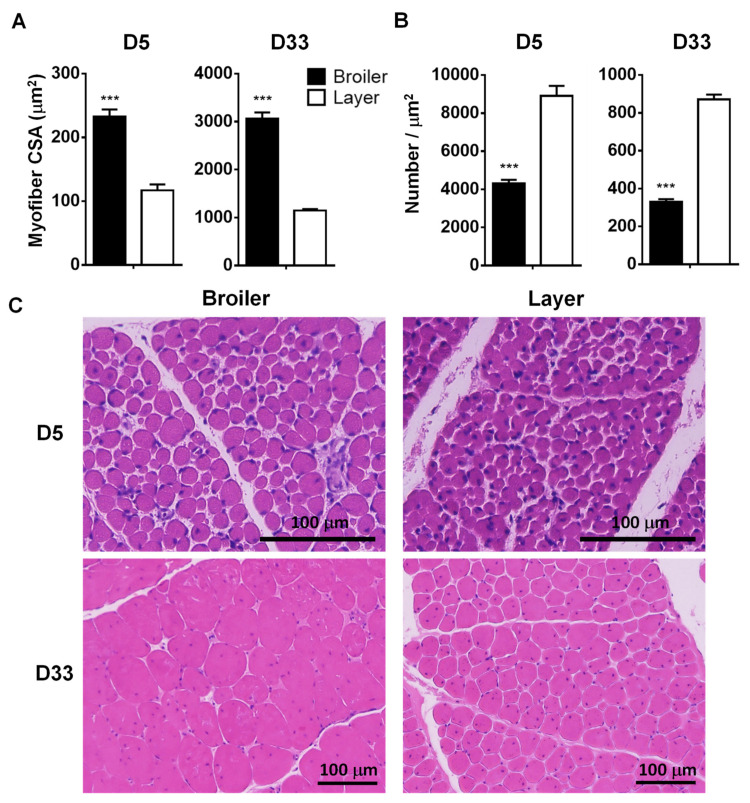
Morphological differences in *pectoralis major* muscles at D5 and D33 between 2 chicken breeds. (**A**,**B**). Comparisons of the cross-sectional area (CSA) of muscle fibers and fiber numbers per unit area. The values are means ± SEM (*n* = 6). The multiple *t*-test was used for statistical analysis of the broiler and layer chickens using the Graphpad PRISM 6.02 program. ***: *p* < 0.001. (**C**). Hematoxylin and eosin staining of pectoralis major muscles. Scale bar: 100 µm. Abbreviations: D, postnatal day.

In the current study, CSA of the muscle fiber in broiler chickens was approximately 2.0 and 2.7 times greater compared to that in layer chickens at D5 (233 ± 11 vs. 117 ± 9 μm^2^, *p* < 0.001) and D33 (3058 ± 114 vs. 1148 ± 30 μm^2^, *p* < 0.001), respectively (Figure 2A,C). In addition, the fiber density per unit area was approximately 2.1- and 2.6-fold greater in the layer compared to the broiler at D5 and D33, respectively (broiler vs. layer: 4301 ± 197 vs. 8907 ± 525, *p* < 0.001 at D5, and 330 ± 14 vs. 871 ± 25, *p* < 0.001 at D33, (Figure 2B,C)), further confirming muscle hypertrophy in broiler chickens.

**Figure 3:**
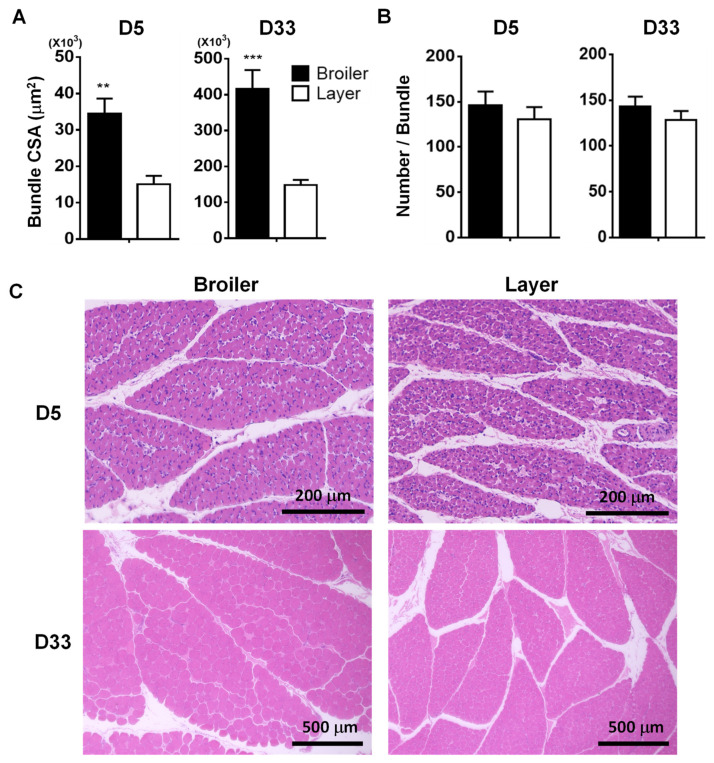
Morphological differences in the bundle of *pectoralis major* muscles at D5 and D33 between two chicken breeds. (**A**,**B**). Comparisons of the bundle CSA and fiber number per bundle. The average of the bundle CSA of PM tissues was calculated by dividing randomly selected areas by the total number of bundles within the area, and over 120 bundles for each animal were measured to calculate the bundle CSA. The values are means ± SEM (*n* = 6). The multiple *t*-test was used for statistical analysis of the broiler and layer chickens using the Graphpad PRISM 6.02 program. **: *p* < 0.01 and ***: *p* < 0.001. (**C**). Hematoxylin and eosin staining of pectoralis major muscles. Scale bar: 200 µm for D5 and 500 µm for D33. Abbreviations: D, postnatal day.

To our knowledge, bundles of muscle fibers (fascicle) have not been characterized regarding the bundle size and fiber number within a bundle in chickens. To compare these bundle characteristics between the broilers and layers, the bundle CSA of PM muscle and fiber numbers in a bundle were analyzed. The current study revealed that broiler chickens showed a larger bundle area compared to layer chickens at D5 (34,450 vs. 15,068 μm^2^, *p* < 0.001) and D33 (415,689 vs. 145,434 μm^2^, *p* < 0.001) (Figure 3A,C). However, the fiber number per bundle was not significantly different between the 2 breeds at both D5 and D33 (Figure 3B,C).

## 4. Discussion

The production of more and leaner meat represents traits that have been pursued by meat producers in the poultry industries. Recent studies demonstrated that MSTN knockout in chickens and quail increased muscle mass [4,5], confirming the anti-myogenic function of MSTN in avian species, which has also been shown in mammals [1,2,3]. The broiler breeds are fast-growing chickens, raised specifically for meat production, and layer breeds are slow-growing chickens, raised for egg production. Therefore, previous research has attempted to link the different muscle characteristics between broilers and layers with the expression levels of *Mstn* in these chickens. A previous study reported that the expression levels of *Mstn* were not different at embryonic ages and post-hatch day 10 between broiler and leghorn chicken breeds [11]. The current study revealed that *Mstn* expression levels were not different between broiler and layer chickens at embryonic ages and post-hatch day 33. However, *Mstn* expression was significantly higher at post-hatch day 5 in broiler chickens compared to layer chickens. Similarly, higher expression of *Mstn* at post-hatch ages in a broiler breed compared to the Wuding chicken breed with a slower growth rate has been reported [12]. Considering the anti-myogenic effect of *Mstn*, the expression levels of *Mstn* are not always negatively correlated with muscle growth, suggesting the existence of possible post-transcriptional regulatory mechanisms of *Mstn*. In fact, avian *Mstn* is found in several mRNA isoforms by alternative splicing mechanisms [6,13]. Therefore, the temporal expression levels of *Mstn* isoforms were the focus of the current study, which compared broiler and layer chickens with distinct muscle growth characteristics. 

The expression patterns of the AS isoforms of *Mstn* vary between different tissues in the same individual poultry with an identical genomic sequence in the whole body [6], suggesting the involvement of other regulatory factors in AS events. Although, in the current study, identical splicing consensus sequences were shown in the broiler and layer chickens, the differences in the ratios of *Mstn-B/-A* expression suggest that exonic/intronic enhancers and silencers [14] might regulate alternative splicing events in the chicken *Mstn* gene. Although the mRNA expression levels of *Mstn* were not different between the two chicken breeds, the different expression ratios of *Mstn-B* and *-A* might contribute to the differences in the growth rates between broiler and layer chicken breeds.

A recent study reported that MSTN knock-out resulted in both muscle hypertrophy and hyperplasia in chickens [5]. Our in vitro studies demonstrated the existence of Mstn isoforms in avian species [6]. Opposite to the anti-myogenic function of the Mstn-A form, the Mstn-B form increased the number of quail myoblasts and enhanced the myotube number and thickness [6]. A pro-myogenic function of the Mstn-B form was confirmed in vivo by the finding that transgenic quail overexpressing the MSTN-B form caused increased muscle mass [7]. It has been reported that both myofiber hypertrophy and hyperplasia are contributing factors to the larger muscles in broilers compared to layers [15]. Therefore, considering the pro-myogenic activities of the *Mstn-B* form and greater muscle accretion with muscle hypertrophy and hyperplasia in broilers [15], the greater ratio of *Mstn-B* to *-A* in broilers compared to layers might be involved in the regulation of muscle growth in chickens. 

In general, fiber numbers per bundle are fixed before or immediately after birth, and an increase in the bundle size is accompanied by an increased muscle fiber size, resulting in subsequent muscle growth [16]. A previous study reported that two broiler breeds exhibited muscle hypertrophy and hyperplasia characteristics in PM tissues compared to the layer at D21 [15]. The current study clearly demonstrated that the larger bundle area in the PM muscle of broilers is caused by increased myofiber size rather than changes in the myofiber numbers per bundle compared to the layers. This new finding in the two chicken breeds needs to be further investigated in other chicken breeds to identify the contribution of myofiber numbers per bundle to muscle growth in chickens. 

Heavy-weight (HW) and low-weight (LW) quail lines have been developed by selectively breeding of more than 80 generations for body weight [17]. The HW quail line showed muscle hypertrophy with a higher ratio of *Mstn-B*/*Mstn-A* forms, and the LW line showed hypoplasia with a lower ratio of *Mstn-B*/*Mstn-A* forms [8,18]. In addition, overexpression of the MSTN-B form in transgenic quail increased muscle mass [7]. Further, overexpression of MSTN-B in the quail cell line caused hypertrophic and hyperplasic myofiber phenotypes [6]. Therefore, given the pro-myogenic potential of the MSTN-B form, it is possible that the higher ratio of *Mstn-B*/*Mstn-A* forms in broiler chickens might partially contribute to muscle growth.

## 5. Conclusions

The characteristics of increased muscle mass and growth rate in broiler chickens are associated with greater muscle bundle CSA due to myofiber hypertrophy. Although in-depth studies are needed to verify the mechanism of this different expressional pattern of MSTN variants between broilers and layers in the future, the current study suggests that *Mstn-B* can serve as a potential positive marker for the selection of superior lines of poultry with enhanced muscle growth. Furthermore, the development of strategies to enhance the expression of the *Mstn-B* form will lead to positive effects in the poultry industry by promoting muscle growth.
